# An Evaluation of Iranian Judges’ Decisions about The Act of Embryo Donation

**DOI:** 10.22074/ijfs.2015.4248

**Published:** 2015-07-27

**Authors:** Alireza Milanifar, Zohreh Behjati Ardekani, Mohammad Mehdi Akhondi

**Affiliations:** 1 Medical Ethics and History of Medicine Research Center, Tehran University of Medical Sciences, Tehran, Iran; 2 Reproductive Biotechnology Research Center, Avicenna Research Institute, ACECR, Tehran, Iran

**Keywords:** Embryo Donation, Law, Iranian, Assisted Reproductive Technology

## Abstract

Embryo donation was one of the infertility treatment methods introduced to the Iranian legal system in 2003 (Act of Embryo Donation) and its by-law passed in 2005 after nu- merous discussions. Embryo donation is a new legal issue in Iran. No similar act has been previously legis- lated in the legal system; however, on the other hand, the importance of the judicial pro- cedure in its execution cannot be ignored since during this treatment process the infertile couples must refer to the court. In this paper, we analyzed 80 court decisions that concerned permission for embryo do- nation during 2006-2011. The decisions were made for couples who requested this treat- ment and referred to Avicenna Fertility Center (Tehran, Iran). In this study, we analyzed the decisions and regulations for the demands, in addition to the medical and legal view- points of the judges. The differences among the judges’ decisions and in the ways of investigating were discussed.

## Introduction

Embryo donation is a method of treatment for couples who lack hope in having a child. In this method, the embryo that arises from other couples’ gametes is transferred to the woman’s uterus for pregnancy and child bearing. The method was introduced to the Iranian legal system in 2003 (Act of Embryo Donation) (1) and its by-law was passed in 2004 following numerous discussions (2).

Sometimes we can find some patients who have come to a clinic 13 times or even more for demanding the treatment (3). In many countries such as Iran, women who cannot have children undergo numerous pressures and threats, along with rejection, family disintegration, cease of financial support and the husband’s remarriage (4). Therefore, although the drugs used in assisted reproductive technology (ART) often have side effects, the patient insists on continuing treatment because of pressures from her husband or relatives.

Gamete and embryo donation, as infertility treatments, bring some moral and legal challenges. Therefore, it is necessary to find moral and legal strategies to inform the society. This is particularly true for families of infertile persons because infertility is a benign, curable disease and not a stigma (3).

Some countries have enacted both similar and dissimilar regulations and rules for embryo donation. Most importantly, embryo or gamete donors and recipients should undergo efficient consultation and be aware of the physical, psychological and legal aspects of embryo donation before making any decision (5). Opposition against organ commercialization and inevitable outcomes of ART such as filiations, rights, and interests of children born from this method has been the impetus for codifying a legal regulation in infertility treatment. Therefore, embryo donation in which a third party is involved can be a suitable alternative for patients. Some countries have special legal provisions for ART, particularly those which involve third parties (6-9).

In Iran, family courts apply different methods for ensuring the competence and qualifications of infertile couples. A lack of unity in the judicial procedure may threaten patients’ best interests. Moreover, inequality before the law is in opposition with article 20 of the Iranian Constitution.

The present study was not conducted about the Act of Embryo Donation or its scientific, moral and legal effects, and results. On the contrary, the significant focus of the study was the demand for embryo donation and the approach adopted by the courts and Iranian legal system in their decision-making process. In the context of existing literature, no similar studies have been performed.

## Materials and Methods

The study was conducted in 80 family courts, which have the authority to permit embryo donation. Their decisions regarding permission for embryo donation during 2006 until the end of 2011 were presented to Avicenna Fertility Center.

Article 8 of the executive by-law regarding embryo donation to infertile couples states that information and documents related to the embryo donation issue are confidential. Moreover, article 648 of the Penal Code of Iran considers the disclosure of medical information and secrets of the patients as a crime. Therefore, for the present study, these decisions have been reviewed by the Clinic Director and all names and numbers were deleted. No data such as claimant’s name, judge’s name and petition number or court branch were mentioned.

The above-mentioned decisions were discussed with regard to claimant, defendant, court location, and male or female infertility factor. The reasons for these decisions were analyzed by taking into consideration the context of the law and its approach towards embryo donation which was legislated in 2003 and the executive by-law (passed in 2005), which have been called "the Embryo Donation Act" and "the regulation" in this paper.

## Results

### Judicial procedure for embryo donation

#### Petition (demand presentation)

According to article 2 of the Act, receiving an embryo should be requested from the court. In the juridical literature, a request or demand is not the same as a petition. In the present study, all 80 mentioned requests were presented to the court in a petition format. Of these, 77 were sent to courts in the centers of provinces and 3 to the courts of small cities.

However, despite the inexplicable nature of this issue, the submission of a petition provides uniform standards and guidelines for the staff.

### The participation of both wife and husband in writing and submitting the request

Article 2 prohibits the wife or husband from separately writing and submitting the request. Infertility treatment by embryo donation needs consent from both the wife and husband. A request from one does not assume any commitment or obligation to the other person according to one of the rules of the Civil Code of Iran (10).

### Right to have a lawyer

According to the Act, both the claimant and defendant have the right to freely hire a lawyer (11). The only condition in embryo donation that could justify the necessity of submitting the request by the couple to the court is the possibility of the court unawareness of the couple’s divorce during the consideration process. However, according to the general context of the law and the term "both wife and husband" where the term "individually" is not mentioned the possibility of submitting the request by a lawyer is undeniable. Different results have been observed in this study. None of the 80 requests presented to the court were submitted by a lawyer. In all requests the plaintiffs undertook this duty personally.

### Claimants

According to the courts’ decisions considered for the claimant it was determined that different people presented as claimants (Table 1).

The 77 claimants in the provincial courts comprised 9 wives, 29 husbands, 38 couples and 1 infertility clinic. Among 3 claimants in the courts of cities, there was 1 wife and 2 couples. Obviously, judges were not unanimous in determining the claimants.

**Table 1:** The parties in the Courts


Location	Lawyer		Claimant				Defendant				
	-	+	Wife	Husband	Both	None	Wife	Husband	Both	None	Court

77	77	0	9	29	38	1	30	8	6	6	27
3	3	0	1	0	2	0	0	0	3	0	0


### Defendants

Based on courts’ decisions, different people were presented as defendants. Among 77 decisions in provincial courts, there were 30 wives, 8 husbands, 6 couples, and 27 Presidents of Family Divisions who were regarded as defendants. In 6 cases, no one was considered to be the defendant. In all 3 cases in the courts of cities, the judge was considered as the defendant. A great disparity existed in determining the defendant.

When the decision was about non-litigious matters, the only requirement was to submit the petition to President of the Family Division or to the prosecuting attorney (12) (Table 1).

### Requirements (conditions)

#### Official ensuring

As previously mentioned, the court is responsible for gathering different types of data about the couples. The adverb "in case of …" insists on the court’s duty because the opposite meaning of the above sentence is that “in the case of not obtaining”, the court will not issue the permission to receive an embryo.

The most important, ambiguous issues in the judicial process of embryo donation are the conditions mentioned above and the way of their assurance.

In order to determine to what extend the court has authority in evaluating the requirements and identifying alternative methods, the conditions and requirements should be differentiated and separately analyzed.

### Infertility and the ability for receiving an embryo

The legal criterion for infertility is an accredited medical certificate. However, there is no accurate definition for the word "accredited". Some issues regarding the impossibility of having a child or the ability of wife to receive an embryo are ambiguous. In this part, we elaborate the issues in detail.

### The accredited medical certificate

In the phrase "accredited medical certificate", the word "accredited" is associated with "certificate" rather than the word "medical". As it is perceived by the phrase, classifying certificates into accredited and non-accredited is not the matter and the actual validity of the certificate is not indicated in its name. A medical certificate issued by an authorized person such as a physician seems to be an accredited certificate.

Therefore, we can conclude that a qualified infertility clinic is the same as an accredited infertility clinic. According to article 1 of the Act concerning medical issues legislated in 1954 and its revisions and amendments that every medical institute shall receive a special license from the Ministry of Health, it can be argued that qualified centers are the same as those holding valid licenses. However, by considering the contexts of the article, the certificates issued by other, unmentioned centers or issued by a physician who is not a member of these centers, are not necessarily invalid. Therefore, the conditions for invalidation of these certificates must be clarified.

From 80 studied decisions, 6 had documented medical certificates from infertility clinics and 42 presented forensic medicine certificates issued by the Legal Medicine Organization of Iran (LMO). A total of 31 decisions documented both certificates and 1 decision documented no certificates (Table 2).

Thus, evaluating the requirements by the court should be based on medical certificates. Referring couples to LMO for confirmation of their condition is not mentioned in the law, even indirectly. This is not a sound decision, which leads to confusion among couples and wastes resources.

**Table 2:** Accreditation authorities


Accreditation	LMO	Infertility clinic	Both	None

Total=80	42	6	31	1


LMO; Legal medicine organization of Iran.

Of note, the release of information such as a medical certificate number or the agent that has issued the permission is against treatment confidentiality

### The ability of wife to receive an embryo and pregnancy

The ability should be considered from two aspects: the physical ability for embryo transfer and the ability for pregnancy and child birth. The first issue mostly depends on the condition of the wife’s uterus whereas the second aspect mostly relates to her body’s general health. The Act used the word "ability" in an absolute sense and does not apply to any adverbs.

Therefore, the important question is whether the woman is capable of receiving an embryo based on the above-mentioned criteria or not. The answer depends on our viewpoint about life principles and its philosophy, a child’s benefits and expediency, and couple’s rights.

Although the conditions mentioned and their associated regulations have been included in the bylaw, a tremendous gap exists in the definitions and the requirements of embryo reception.

There is a defensible argument that the wife is able to receive the embryo regardless of medical criteria. Basically, a wife must have the capability to become pregnant. Thus an older woman is excluded from this law even if she can meet the necessary medical requirements. This is closer to the expediency of the child (13).

### Proven infertility

As considered in article 1 of the Act, infertility of one or both members of the couple should be clearly proven; though determining incurable infertility is not an easy task.

Infertility is clearly defined in medicine. Infertility refers to the fact that a couple with the aim of having a child is not able to conceive after one year of unprotected sexual intercourse (14). An important point according to the definition of infertility is whether the couple is capable of receiving an embryo or not. In other words, whether the infertility of the couple is sufficient documentation or whether it is necessary for the couple to prove their attempts for treatment and ultimate failure.

As understood from the last part of Article 1, infertile couples should receive medical treatments and according to the article, their infertility must be proven. However it is not mentioned how many times the couples must attempt and whether there is any benefit in obliging them to treat their infertility.

Certainly, in some cases medical treatment can be continued for a long time but it seems that the couple cannot be forced to accept treatment when there is no chance for success with treatment. The side effects of these treatments are indisputable. Therefore, we can claim that the context of the law in terms of this matter is not sufficiently clear.

Of note, the use of ART by postmenopausal women is a matter of controversy. Opponents, by citing the principle of non-malfeasance, believe in high risk of pregnancy of older women. By referring to the principle of beneficence, they believe in the best interests of the child with regards to the high possibility of losing his/her mother at a younger age. However, proponents resort to the principle of justice and strongly advocate the continuation of this right. Younger women are prioritized when an appropriate budget is available (15, 16). There is no explicit article about these types of women in the Act; only article 7 of the executive by-law about embryo donation asks for a certificate that pertains to the ability of the wife for pregnancy and receiving the embryo.

Among 80 reviewed decisions, 70 were related to male infertility and the remaining 10 were related to female infertility. There was no significant difference between provinces and cities. The large difference between the two above-mentioned numbers showed that most cases of infertility which resulted in embryo donation were due to male factor, an important finding which should be investigated thoroughly. Perhaps fertile men could provide an alternative for their infertile wives (by remarriage or polygamy), an opportunity which was unachievable for women. Based on these arguments, further research is needed in this area.

### Moral competence

Moral competence shall be determined by the court. In Iranian legal literature, there is no definition of moral competence. In certain cases, instances of incapacity have been noted, such as corruption, drug addiction or previous criminal convictions (article 14 of the Act of Governmental Employment and article 1173 of Civil Code of Iran).

There are two factors to be considered with respect to moral competence. First, the instances of capacity and incapacity and secondly, the way of determining them should be identified. However, these issues have not been included in the Act.

The 80 decisions studied showed that the courts did not mention what should be determined. Mostly they considered the method (how to) of determination.

Among the 80 studied decisions, 14 were cited by local researches, 1 was cited by a police report, 13 were cited by local affidavits and 26 were related to the principle of validity (omnia praesumuntur rite esse acta).

In 24 cases, it was only mentioned that the court determined the general competence of the couple. In 2 cases, there was no mention of the couple’s competence (Table 3).

A few judges made use of external assistance for determining competence. However, others believed that they did not need to resort to external help.

Among 77 cases referred to regional courts, in 13 the judges pursued local investigation and in 64, they did not.

Among 3 cases referred to city courts, in 1 local investigation was used as the main method for determining moral competence and in 2, this approach had no place in the judges’ decisions.

Some judges have considered accuracy as the main principle for moral competence determination. However, this principle is more applicable in a juridical act and contract, yet not in determining an existing issue. Among 77 cases which had been referred to the provincial courts, in 26 the principle was included in the process of decision making.

None of the 3 cases referred to the city courts had the accuracy principle included.

Among 77 cases referred to the provincial courts, in only 1 case an inquiry by the police helped the judges to determine the couple’s moral competence. In 76, the judges did not rely on this method. None of the judges inquired from the police in 3 cases which had been referred to the city courts. In all cases, none of the judges accepted the principle of innocence.

Most judges used certain methods which were against the principles of confidentiality. For example, among 77 cases which had been referred to the provincial courts, in 14 a local affidavit was used. In 63, this method was not used.

Among 3 cases which had been referred to the city courts, in 1 the judge relied on local investigation whereas in the other 2 cases the judges did not adopt this approach.

Among 15 cases in which local research was used, the infertility reason for all cases was male factor.

**Table 3:** Instances of capacity


Instances of capacity	Local investigation	Police	Principle of validity	Testimony	Mentioned	Not mentioned

80	14	1	26	13	24	2


Among 65 cases in which a local research method was not used, the infertility reason in 55 cases was male factor and for 10 cases, it was female factor.

### Iranian nationality of the couple

According to article 976 of the Civil Code of Iran, a native Iranian is someone whose father is Iranian or who holds an Iranian citizenship by one of the mentioned ways in the law. The wife of an Iranian man is considered a native Iranian as long as she is not separated or divorced. Birth and marriage certificates can be considered documents that prove Iranian nationality.

## Conclusion

According to the aforementioned points, the enactment and legal issues in embryo donation remain unknown among lawyers and judges. This can be attributed to a lack of an identical judicial process for legal procedures and non-interference by lawyers in judicial dossiers.

The legal issues in embryo donation are mostly studied at the law school level which may be a cause for various judicial processes. For this, it is necessary to consider the following points:

Embryo donation as a treatment method for infertile couples must be carried out exclusively by infertility clinics that have sufficient capabilities. The possibility of carrying out this treatment must be studied separately for each couple. Issuing a certificate based on confirming general physical and mental health of the couple is one of the tasks of treatment centers. This certificate shall be offered to the legal authority (the president of the court) as an attachment with the couple’s personal request or by a lawyer.

Obtaining good standing certificate records can result in unity in the judicial procedure.

The court’s avoidance from performing local investigations-developing a local affidavit or demanding testimony, researches by local police or publishing the court’s decision in more than 1 copy all of which oppose the confidentiality of treatment- are important points that should be taken into consideration. Familial relationships in small cities and towns have conflicts with medical confidentiality. Due to couples’ tendency keep silent about the treatment; it is appropriate to allocate special branches of courts in large cities to prevent such conflicts. Educating the staff and judges about the bases of the treatment and its medical, legal, ethical and social aspects would be beneficial.

The court’s decision for permitting embryo donation (transfer of donated embryos) should be limited to a definite period of time (e.g., 1 year).

Of note, permission given by the court does not obligate the infertility clinic to perform an embryo donation. Although the clinic should determine the basic capacities and facilities necessary for issuing the certificate to the court, this cannot inhibit these centers from using new methods or additional studies. A physician considers his/her colleagues’ opinions about a patient as useful advice and not mandatory orders. Therefore, it is not surprising that the opinion of a physician or a medical team for determining the couple’s competence may be considered wrong and unsound by another medical team (even if it leads to the court’s permission for receiving a donated embryo).

Couples are recommended to follow their request process from the first step in the infertility clinic to which they will refer after the court’s permission, in order to respect their physician’s diagnosis and treatment, irrespective of the practices and opinions of other physicians.
